# A practical assessment protocol for clinically relevant P-glycoprotein-mediated drug-drug interactions

**DOI:** 10.3389/fphar.2024.1412692

**Published:** 2024-09-30

**Authors:** Leonie Bogaard, Kayan Tsoi, Bas van de Steeg, Esther F. A. Brandon, Lisanne Geers, Margreet van Herwaarden, Frank Jansman, Dominique Maas, Margje Monster-Simons, David S. Y. Ong, Sander D. Borgsteede

**Affiliations:** ^1^ Department Clinical Decision Support, Health Base Foundation, Houten, Netherlands; ^2^ Department of Clinical Pharmacy, Canisius Wilhelmina Hospital, Nijmegen, Netherlands; ^3^ Dutch Medicines Evaluation Board, Utrecht, Netherlands; ^4^ Department of Clinical Pharmacy, Rijnstate Hospital, Arnhem, Netherlands; ^5^ Community Pharmacy Groesbeek, Groesbeek, Netherlands; ^6^ Unit of PharmacoTherapy, Epidemiology and Economics, Groningen Research Institute of Pharmacy, University of Groningen, Groningen, Netherlands; ^7^ Department of Clinical Pharmacy, Deventer Teaching Hospital, Deventer, Netherlands; ^8^ Department of Internal Medicine, Radboud University medical center, Nijmegen, Netherlands; ^9^ Department of Clinical Pharmacy and Pharmacology, University of Groningen, Groningen, Netherlands; ^10^ Department of Medical Microbiology and Infection Control, Franciscus Gasthuis and Vlietland Hospital, Rotterdam, Netherlands; ^11^ Department of Epidemiology, Julius Center for Health Sciences and Primary Care, University Medical Center Utrecht, Utrecht University, Utrecht, Netherlands

**Keywords:** drug-drug interaction, P-glycoprotein, practice recommendations, literature review, expert opinion, study protocol, clinical decision support

## Abstract

**Background:**

Drug-drug interactions (DDIs) may influence the effectiveness and safety of medication treatment, which may require additional monitoring, dose adjustment or avoidance of certain drugs. DDIs involving P-glycoprotein (P-gp) affect many drugs, but current official product information is often insufficient to guide the management of these DDIs in clinical practice. The aim of this paper is to describe a protocol to assess DDIs involving P-gp and to develop and implement practice recommendations for clinically relevant P-gp-mediated DDIs that affect clinical outcomes through changes in systemic drug exposure.

**Methods:**

A combined literature review and expert opinion approach will be used according to the following seven steps: set up an expert panel (step 1), establish core concepts and definitions (step 2), select potential P-gp-modulators (i.e., P-gp-inducers and -inhibitors) and P-gp-substrates to be evaluated (step 3), select and extract evidence-based data, and present findings in standardized assessment reports (step 4), discuss and adopt classifications and practice recommendations with the expert panel (step 5), publish and integrate information and alerts in clinical decision support systems (CDSS) (step 6), (re)assessments of DDIs and potential new DDIs when new information is available or when initiated by healthcare providers (step 7).

**Anticipated results:**

The expert panel will classify potential P-gp-modulators and -substrates as clinically relevant P-gp-inducer, -inhibitor and/or -substrate and draw conclusions about which combinations of classified modulators and substrates will lead to clinically relevant DDIs. This may include the extrapolation of conclusions for DDIs where limited or no data are available, based on the pharmacological characteristics of these drugs. For (potential) DDIs that are considered to be clinically relevant, practice recommendations will be developed.

**Discussion:**

This protocol describes a standardized, evidence- and expert opinion-based assessment of P-gp-mediated DDIs that affect clinical outcomes. This approach will generate alerts with practice recommendations for clinically relevant DDIs and transparent rationales for DDIs that are considered to be irrelevant. These recommendations will improve individual patient care by supporting healthcare professionals to make consistent decisions on how to manage P-gp mediated DDIs.

## Introduction

P-glycoprotein (P-gp) is recognized as an efflux transporter that can play a role in clinically relevant drug-drug interactions (DDIs), by affecting the bioavailability (i.e., systemic exposure) of exogenic substances such as medication (ICH, 2022; Zamek-Gliszczynski et al., 2021). P-gp is also known as multidrug resistance protein (MDR1) and adenosine triphosphate (ATP) binding cassette sub-family B member 1 transporter (ABCB1) (Taskar et al., 2022). It is expressed in various tissues with an excretory function, including the intestine, liver, kidney and brain (Taskar et al., 2022; Elmeliegy et al., 2020; Lin and Yamazaki, 2003). P-gp plays a role in the excretion of foreign substances into the intestinal lumen, bile and urine, and in preventing accumulation in the brain (Taskar et al., 2022; Elmeliegy et al., 2020; Lin and Yamazaki, 2003). Changes in systemic drug exposure due to DDIs involving P-gp are mostly related to modulation of P-gp in the intestine, as most drugs primarily modulate intestinal P-gp (Zamek-Gliszczynski et al., 2021; Taskar et al., 2022; Elmeliegy et al., 2020). There is limited clinical relevance for DDIs due to P-gp-inhibition in the liver and kidney, and P-gp appears to be less sensitive to induction in the liver and kidney (Zamek-Gliszczynski et al., 2021; Taskar et al., 2022; Elmeliegy et al., 2020). Although P-gp is known to be involved in drug penetration into the brain, most scientific knowledge on the DDIs with P-gp are regarding intestinal DDIs (ICH, 2022; Zamek-Gliszczynski et al., 2021; Kalvass et al., 2013). Therefore, this review will focus on changes in systemic drug exposure involving modulation of P-gp in the intestine.

From a pharmacological perspective, accurate interpretation of the apparent P-gp-effect is challenging due to the diversity of P-gp-expression and overlap with other transporters and drug metabolizing enzymes (ICH, 2022; Zamek-Gliszczynski et al., 2021; Elmeliegy et al., 2020; Lin and Yamazaki, 2003; Biotechnology, 2024; Tornio et al., 2019; Zhang et al., 2018). It can be difficult to distinguish different pharmacological mechanisms as many P-gp-modulators modulate not only P-gp but also other transporters and/or CYP enzymes, such as CYP3A4 (ICH, 2022; Amin, 2013; Wang et al., 2012; Umeyama et al., 2014). In addition, few selective P-gp-modulators and substrates are known (ICH, 2022; Elmeliegy et al., 2020; Lin and Yamazaki, 2003; Biotechnology, 2024; Tornio et al., 2019; Zhang et al., 2018).

The U.S. Food and Drug Administration (FDA), European Medicines Agency (EMA) and The International Council for Harmonisation of Technical Requirements for Pharmaceuticals for Human Use (ICH) have published (draft) research guidelines for investigating the potential role of P-gp in new drug applications (ICH, 2022; Lin and Yamazaki, 2003; FDA, 2020b; EMA, 2012; del Amo et al., 2009). The research guidelines recommend that investigational drugs should first be assessed *in vitro* for substrate or inhibition characteristics, and provide guidance on when to consider clinical studies (FDA, 2020b; EMA, 2012). *In vitro* methods to assess P-gp-induction are not well established (ICH, 2022; Elmeliegy et al., 2020; FDA, 2020b; FDA, 2020a; Lin, 2007). The primary purpose of clinical studies during drug development is to determine the anticipated largest magnitude of DDIs and not to gain a (quantitative) mechanistic understanding, or to investigate all possible DDIs (ICH, 2022; Elmeliegy et al., 2020; FDA, 2020a). Moreover, it is not feasible to study all possible DDIs (ICH, 2022; FDA, 2020a). In the case of a new P-gp-modulator, it is often unclear how it will affect P-gp-substrates that have not been studied. In the case of a new P-gp-substrate, often only interaction with a single P-gp-modulator has been studied and it is not clear how other modulators will affect this substrate.

Despite these guidelines, current official product information often contains inconclusive and inconsistent recommendations on P-gp-mediated DDIs (Burger et al., 2023; Gessner et al., 2019). Patient safety could be at risk if potential DDIs are not listed in the product information (lack of sensitivity) or if recommendations lack clinical relevance (lack of specificity), potentially depriving patients of the best available therapy (Lin and Yamazaki, 2003; del Amo et al., 2009; Henderson et al., 2021; van der Sijs et al., 2006). It is therefore valuable to support healthcare professionals with sensitive and specific alerts and practice recommendations, including suggestions for avoiding certain combinations, adjusting the dose or monitoring outcomes such as plasma levels or clinical effects, as well as transparent rationales for DDIs that are considered to be irrelevant. These recommendations should be based on the available evidence and expert opinion in the context of the potential clinical relevance and consequences of P-gp-mediated DDIs for the individual patient. To ensure consistency of quality, it is important that the method for assessing P-gp-mediated DDIs and developing practice recommendations is standardized and transparent (Floor-Schreudering et al., 2014).

Similar evaluations have been successfully carried out for pharmacological challenges such as DDIs in oncology or COPD (van Leeuwen et al., 2022; Wang et al., 2019), the safe use of medication in patients with cirrhosis (Weersink et al., 2016), or the management of drug-disease interactions and DDIs (van Tongeren et al., 2020; van Roon et al., 2005) The aim of this paper is to describe a transparent and standardized protocol to systematically develop evidence and expert opinion-based practice recommendations for P-gp-mediated DDIs that affect treatment outcomes, including their implementation in clinical decision support systems (CDSS) for healthcare professionals to improve patient care.

## Methods

The development and implementation of practice recommendations according to seven steps will be described in detail below and illustrated with a flow chart in the Supplementary Material 1.

### Step 1: Setting up an expert panel

All steps in this protocol will be performed by two pharmacist-reviewers (LB, KT), experts in the evaluation of the safety of drugs under supervision of a clinical pharmacologist/epidemiologist (SB) and a multi-disciplinary expert panel. Both reviewers are trained in literature extraction and quality assessment. The expert panel consists of healthcare professionals with knowledge of P-gp as pharmacological mechanism from different perspectives: two prescribers (internal medicine and clinical microbiology), four pharmacists (two community pharmacists, and two hospital pharmacists/clinical pharmacologists) and five members with expertise in the evaluation of safety and pharmacology (two clinical assessors from the Medicines Evaluation Board with expertise in assessing and interpreting DDI studies, and three assessors from the Health Base Foundation, with experience in translating pharmacological data into clinical actions).

The role of the expert panel involves five subsequent steps of the protocol including determination of the core concepts and definitions (Step 2), the selection of potential P-gp-modulators and -substrates to be evaluated (Step 3), literature selection criteria (Step 4), and discussing classifications and practice recommendations (Step 5). If necessary, healthcare professionals with expertise in a specific condition will be asked for advice for their therapeutic areas. Conclusions will be based on consensus and potential conflicts of interest of the expert panel will be identified and published.

### Step 2: Concepts and definitions

To develop a consistent and transparent protocol, the following aspects will be considered: the use of human *in vivo* data, no-effect boundaries, P-gp-induction predicted by CYP3A4-induction, concepts about the magnitude of P-gp modulation and definition of (potentially) clinically relevant effects.

#### Human *in vivo* data

The focus will be on human *in vivo* studies, as this is rated more convincing than evidence from *in vitro* studies to determine the clinical relevance of P-gp-mediated DDIs (ICH, 2022; FDA, 2020b; EMA, 2012; Kido et al., 2024). As described by the ICH-M-12 guidelines, various *in vitro* transporter assays can be used to screen the risk for transporter-mediated interactions when appropriate positive controls and reference substrates are included in the test (ICH, 2022). *In vitro* studies can indicate if and which further clinical studies need to be conducted to quantify the magnitude of these DDIs or to conclude about the clinical relevance (ICH, 2022; del Amo et al., 2009; Lin, 2007). In case of limited or inconsistent data from human *in vivo* studies, *in vitro* data may be included to give a mechanistic understanding and to evaluate confounding mechanisms, e.g., other transporters or drug metabolizing enzymes. Animal studies are not included because they are also difficult to translate to humans and clinical relevance (ICH, 2022; FDA, 2020b; EMA, 2012; Kido et al., 2024). In addition, animal studies are also not commonly used to study DDIs in drug development because these data are not required by the regulatory authorities (ICH, 2022). In the absence of clinical studies, Physiologically Based PharmacoKinetic (PBPK) models can be included to predict the DDI risk of a drug, but the translation of these models into the clinical setting is not yet fully established and should be evaluated on a case-by-case basis (Taskar et al., 2022; Rowland Yeo et al., 2024). In general, pharmacogenetic studies can sometimes complement or replace DDI studies. A mechanistic understanding of the pathway for the PK of the investigational drug can best be studied when genetic variability results in loss-of-function or altered activity (Tornio et al., 2019). Although numerous studies have attempted to find associations between various single nucleotide polymorphisms of the ABCB1 gene and drug PK, the results are variable and conflicting (Saiz-Rodríguez et al., 2018). More knowledge is needed on this topic to assess the pharmacogenetic effect in relation to DDIs involving P-gp. Therefore, genetic-based studies will not be included as evidence.

#### No-effect boundary of 80 to 125 percent

As defined by the FDA and EMA in bio-equivalence studies, a default no-effect boundary of 80 to 125 percent will be used: if the measured change in systemic exposure (AUC) in the DDI study falls within this chosen interval, the effects of a potential P-gp-modulator or substrate is considered to be not clinically relevant (ICH, 2022; EMA, 2012; FDA, 2020a). An exception will be made for drugs classified as high-risk P-gp substrates based on drug-specific characteristics (e.g., narrow therapeutic index drugs) or expert opinion (see *“definition of (potentially) clinically relevant effects”* below). For these drugs the no effect boundary will not always be applicable (EMA, 2010).

#### P-gp-inducers predicted by CYP3A4-induction

Established strong and moderate CYP3A4-inducers will be evaluated as potential P-gp-inducers (see also Step 3) (ICH, 2022). P-gp-induction is regulated by transcriptional activation by nuclear receptors, such as the pregnane xenobiotic receptor (PXR) and the constitutive androstane receptor (CAR). Both PXR and CAR can regulate the expression of many enzymes such as CYP3A4, and CYP3A4 induction is predictive of P-gp-induction (ICH, 2022; Wang et al., 2012; Lutz et al., 2018). However, weak CYP3A4 inducers (AUC decrease of a sensitive probe substrate < factor 0.5) are not expected to elicit relevant P-gp-induction, since P-gp is less inducible than CYP3A4 (ICH, 2022; Zamek-Gliszczynski et al., 2021; Elmeliegy et al., 2020). CYP3A4-inhibition has not been described as a predictor of P-gp-inhibition. Therefore, CYP3A4-inhibitors will not be considered as potential P-gp-inhibitors based solely on their CYP3A4-activity.

#### The magnitude of the effect of a P-gp-inducer or -inhibitor will not be classified

The available evidence is insufficient to classify the magnitude of the effect of a P-gp-modulator and the magnitude of the DDI-effect mediated by transporters has a limited range (ICH, 2022; Elmeliegy et al., 2020).

#### Definition of (potentially) clinically relevant effects

In general, P-gp-mediated DDIs can result in increased plasma levels with a risk of adverse drug events and toxicity when substrates are combined with inhibitors due to increased absorption, or decreased plasma levels with potentially reduced treatment efficacy when combined with inducers due to decreased absorption. For each potential substrate, the clinical relevance of the results of the DDI studies will be assessed as “yes”/“no”/“uncertain”, based on available pharmacokinetic evidence (e.g., AUC/C_max_) and clinical risk evidence (e.g., toxicity/reduced treatment efficacy) (ICH, 2022). In general, the clinical relevance of potential DDIs (i.e., the need for extrapolation) will be different for substrates with a high risk of toxicity or reduced treatment efficacy, depending on the therapeutic window. These drugs are often referred to as narrow therapeutic index drugs. Therefore, substrates will be assessed as *high risk P-gp-drug-substrates* ‘yes’ if substrates meet at least one of the following criteria:

1. Difference between toxic dose compared to effective dose is ≤ factor 2 (Elyes Dahmane, 2024; Yu et al., 2015);
2. Small differences in dose (≤ factor 2) or blood concentration may lead to serious therapeutic failures and/or toxicity (Elyes Dahmane, 2024; Yu et al., 2015);
3. Low within subject variability (≤30%) for AUC and/or C_max_ (Elyes Dahmane, 2024; Yu et al., 2015);
4. Drugs that are dosed individually due to clinically relevant inter-individual variability and/or with Therapeutic Drug Monitoring (TDM), and for which doses are often adjusted in small increments (<20%) (Elyes Dahmane, 2024; Yu et al., 2015; Tyson et al., 2020);
5. One of the following (classes of) drugs: antineoplastic agents (ATC L01A*-L01D*), immunosuppressants for transplant rejection (ATC L04B*), anti-infective drugs (ATC J*), antiepileptic drugs (ATC N03A*), anti-thrombotic drugs (B01A*) and drugs that can easily lead to severe intoxications (e.g., lithium, QT prolonging drugs based on CredibleMeds QTDrugs Lists, calcium-channel-blockers, TCAs, MAOs) (Woosley and Romero, 2013; World Health Organization, 2022).

If substrates do not meet any of these criteria, this will result in the outcome *high risk P-gp-drug-substrates* “no.” These criteria will be applied according to the flow chart as shown in Supplementary Material 2.

### Step 3: Selection of drugs to be evaluated

The expert panel will select all drugs for which the potential influence of P-gp is mentioned in any of the following sources:

• Official product information (*SmPC)* and European Public Assessment Reports (EPAR). For all drugs available in the Netherlands, all SmPCs and U.S. FDA drug labels that mention (potential) DDIs with P-gp will be evaluated. The following sections in the SmPC and the FDA label information (highlights of prescribing information) will be searched for relevant information about DDIs: sections 4.3/4.4/4.5 and sections 4/5/7, respectively.
• Drugs mentioned in the review about P-gp-interactions by Lund et al. as clinically relevant P-gp-modulator or -substrate (Lund et al., 2017).
• Guidelines and handbooks for DDIs. Drugs that are listed as P-gp-inducer, inhibitor or substrate in one of the following international and national sources: EMA/FDA/ICH-M-12 guidelines, Stockley’s, the EHRA-guideline, Up-to-date and the two Dutch Guidelines for DDIs *Commentaren Medicatiebewaking* and *G-Standaard* (ICH, 2022; FDA, 2020b; EMA, 2012; Steffel and Heidbüchel, 2021; Baxter, 2024; G-standaard - Medicatiebewaking, 2024; Post, 2024; Commentaren Medicatiebewaking - Interacties, 2024). In addition, all drugs that are listed by the FDA as strong/moderate CYP3A4-inducers (see Step 2) (FDA, 2024).

### Step 4: Selection and presentation of evidence

For each potential P-gp-inducer, -inhibitor and/or -substrate selected in Step 3, additional evidence will be collected by a systematic literature review. The electronic databases PubMed and EMBASE will be searched by the search strategy outlined in Table 1 and available in the assessment reports (see Supplementary Material 3). Additional publications will be added manually by citation tracking. The collection of evidence in this paper will adhere to the PRISMA-P (Preferred Reporting Items for Systematic review and Meta-Analysis Protocols) checklist for systematic review protocols (See Supplementary Material 4) (Moher et al., 2015) Also, the level of evidence of each study will be assessed according to the criteria for treatment harms of the Oxford Centre for Evidence-Based Medicine (CEBM) (OCEBM Levels of Evidence Working Group, 2011). The OCEBM-criteria are a pragmatic heuristic for assessing the quality of studies across the entire range of clinical questions. These criteria have been applied in other studies, such as in recommendations for the safe use of medication in patients with cirrhosis (Weersink et al., 2016; OCEBM Levels of Evidence Working Group, 2011).

**TABLE 1:** Proposed search strategy for PubMed and Embase.

Database	Search query
Pubmed	(“modulator*” [mesh] OR “modulator*” [TIAB]) AND (“Dabigatran” [Mesh] OR “Dabigatran” [TIAB] OR “edoxaban” [Supplementary Concept] OR “edoxaban” [TIAB] OR “Digoxin” [Mesh] OR “Digoxin” [TIAB] OR “fexofenadine” [Supplementary Concept] OR “fexofenadine” [TIAB] OR “talinolol” [Supplementary Concept] OR “talinolol” [TIAB] OR “aliskiren” [Supplementary Concept] OR “aliskiren” [TIAB] OR (ATP Binding Cassette Transporter, Subfamily B, Member 1 [Mesh]) OR (P-Glycoprotein [TIAB]) OR (P Glycoprotein [TIAB]) OR (P-gp [TIAB]) OR (ABCB1 [TIAB]) OR (MDR1 [TIAB])
	(“substrate*” [mesh] OR “ substrate*” [TIAB]) AND (rifampin [Mesh] OR rifampicin [TIAB] OR Carbamazepine [Mesh] OR Carbamazepine [TIAB] OR Phenytoin [Mesh] OR Phenytoin [TIAB] OR apalutamide [Supplementary Concept] OR apalutamide [TIAB] OR efavirenz [Supplementary Concept] OR efavirenz [TIAB] OR Hypericum [Mesh] OR Hypericum [TIAB] OR “Amiodarone” [Mesh] OR “Amiodarone” [TIAB] OR “ranolazine” [Mesh] OR “ranolazine” [TIAB] OR “Quinidine” [Mesh] OR “Quinidine” [TIAB] OR “Propafenone” [Mesh] OR “Propafenone” [TIAB] OR “Cyclosporine” [Mesh] OR “Cyclosporine” [TIAB]) OR (“ATP Binding Cassette Transporter, Subfamily B, Member 1” [Mesh]) OR (P-Glycoprotein [TIAB]) OR (“P Glycoprotein” [TIAB]) OR (P-gp [TIAB]) OR (ABCB1 [TIAB]) OR (MDR1 [TIAB])
Embase	Modulator*/mj AND (‘dabigatran’/mj OR ‘edoxaban’/mj OR ‘digoxin’/mj OR ‘fexofenadine’/mj OR ‘talinolol’/mj OR ‘aliskiren’/mj OR ‘abc transporter subfamily b’/mj OR ‘multidrug resistance protein 1’/mj)
	Substrate*/mj AND (‘rifampicin’/mj OR ‘carbamazepine’/mj OR ‘phenytoin’/mj OR ‘apalutamide’/mj OR ‘efavirenz’/mj OR ‘amiodarone’ OR ‘ranolazine’/mj OR ‘propafenone’/mj OR ‘quinidine’/mj OR ‘cyclosporine’/mj)OR ‘abc transporter subfamily b’/mj OR ‘multidrug resistance protein 1’/mj)

* For each search the name of the specific modulator/substrate will be filled out.

#### Selection of evidence

The selection of evidence will be different for the evaluation of P-gp-modulators and -substrates. The following literature selection criteria will be used.

• Evidence for the evaluation of P-gp-modulators. The evidence will be limited to P-gp-substrates that are described in the EMA/FDA and ICH-M-12 guidelines and that are not metabolized by CYP3A4. Therefore, data of DDIs with the following probe substrates will be used to classify potential P-gp-modulators: digoxin, dabigatran etexilate, edoxaban and fexofenadine (ICH, 2022; EMA, 2012; Lund et al., 2017; FDA, 2024). In addition, talinolol and aliskiren will be considered as probe substrates as they have been established as P-gp-substrates in clinical DDI-studies (Elmeliegy et al., 2020; Tornio et al., 2019; Lund et al., 2017; Baxter, 2018). Other substrates lack selectivity, making accurate interpretation of study results regarding apparent P-gp-effects unreliable.
• Evidence for the evaluation of substrates. For a consistent approach and in the absence of established P-gp probe modulators, potential P-gp-substrates will be evaluated with a selection of P-gp-inducers and P-gp-inhibitors. We defined rifampicin, apalutamide, carbamazepine, efavirenz, phenytoin and St John’s wort as established P-gp-inducers (ICH, 2022; FDA, 2020a; FDA, 2024). The selection of P-gp-inhibitors is limited to inhibitors with no or weak CYP3A4-inhibiting effects: amiodarone, cyclosporine, propafenone, ranolazine and quinidine as P-gp-inhibitors (ICH, 2022; FDA, 2024). In order to distinguish between different pharmacological mechanisms, the metabolism profile of the potential P-gp-substrate in combination with the specificity of the used P-gp-modulators will be taking into account.

#### Data extraction

Table 2 summarizes the data to be extracted with a focus on qualitative data about clinically relevant changes in systemic drug exposure. Data will be presented in separate standardized assessment reports for inducers, inhibitors and substrates (see Supplementary Material 3). According to the data, the pharmacist-reviewers will independently assess the robustness of the available evidence and propose an initial classification of potential P-gp-modulators and -substrates as clinically relevant P-gp-inducer, -inhibitor and/or -substrate (see Table 2 for possible classifications). P-gp-modulators and -substrates will be classified as “established” if there is sound evidence that the drug affects/is affected by P-gp-modulation, as “not relevant” if these effects are considered to be irrelevant or as “unclear” if the data are conflicting. P-gp-inhibitors and -substrates will be classified as “lack of evidence” if there are no human *in vivo* studies or if the interpretation of study results regarding apparent P-gp-effects is unreliable. Due to the described overlap between P-gp- and CYP-induction, P-gp-inducers predicted by CYP3A4-induction will be classified as “predicted P-gp-inducer” in the absence of human *in vivo* studies.

**TABLE 2:** Data extraction and classification of P-gp-inducers, -inhibitors and -substrates.

Data extracted from literature	
• Study characteristics: study type, level of evidence, number of patients and drug regimen.	
• Effects of the inducer/inhibitor on the AUC and C_max_ of the substrate, expressed as increase/decrease (factor) compared to the use without the inducer/inhibitor.	
• Effects on clinical outcomes (effectiveness and ADRs) as mentioned in the specific studies.	
• Any relevant remarks, e.g., on the underlying pharmacology, strengths/limitations of the study.	
• *In vitro* data about the underlying pharmacology if considered relevant in the context of evaluation.	
• Motivation to classify potential substrates as a *high risk P-gp-drug-substrate*, as defined in Step 2 (*only for substrates*).	

Classification of the influence of P-gp of potential P-gp-inducers, -inhibitors and -substrates	
Established	Evidence from human *in vivo* studies of P-gp-induction (AUCR <0.8) AND/OR inhibition (AUCR ≥ 1.25) on substrates.
Predicted *(only for inducers)*	Established strong/moderate CYP3A4-inducers, but lack of human *in vivo* studies with P-gp probe substrates that will classify them as established or as not relevant.
Not relevant	Lack of P-gp-induction (AUCR ≥ 0.8) AND/OR inhibition (AUCR < 1.25) on substrates in human *in vivo* studies.
Unclear	Inconclusive evidence from human *in vivo* studies, e.g., where some combinations result in an interaction-effect, while no effect with other combinations.
Lack of evidence	Lack of evidence from human *in vivo* studies.

### Step 5: Conclusion and practice recommendations for clinical practice

The assessment reports generated in Step 4 will be discussed with the expert panel. The panel will determine the final classification of P-gp-modulators and/or -substrates, the robustness of the evidence, the clinical relevance of the results of DDI studies, and whether the substrate has a high risk of toxicity or reduced treatment efficacy.

The combination of classified P-gp-modulators and -substrates will result in the following conclusions: a drug will be involved in clinically relevant P-gp-interactions (yes or no). This may include the extrapolation of conclusions for DDIs for which limited or no data are available, based on the pharmacological characteristics of these drugs. For (potential) DDIs that are considered to be clinically relevant, practice recommendations will be developed with specific actions, i.e., to avoid certain combinations, adjust the dose or monitor outcomes such as plasma levels or clinical effects. Each discipline should be able to apply the recommendations in their clinical context.

The expert panel will reach conclusions by consensus and this will be included in the assessment reports. Differences of opinion within the expert panel will be added as comments.

### Step 6: Implementation in electronic prescribing and dispensing systems

The assessment reports will be published in the Dutch guideline for DDIs (Commentaren Medicatiebewaking), and the recommendations will be implemented in Pharmabase, one of the two National Drug Databases in the Netherlands. This will guarantee that the outcomes will be adopted in Dutch Healthcare Information Systems supporting prescribing and dispensing processes, which is used in about 65% of community pharmacies and 45% of the general practices (PharmaPartners, 2018; SFK, 2008; PW, 2023). Whenever interacting drugs will be prescribed/dispensed together, an alert will be generated. Relevant background information concerning the advices will be accessible and referring to the standardized assessment reports. To support international dissemination, the results will be published as summary tables of classified modulators and substrates including recommendations in international, peer-reviewed, scientific journals.

### Step 7: Updating

The following three maintenance activities will keep the assessment reports, related advices and background information up-to-date:

1. New drugs marketed in the Netherlands will be screened monthly to identify drugs to be submitted in Step 3 of the assessment procedure.
2. In case of new information identified by regulatory authorities (Dutch Medicines Evaluation Board, European Medicines Agency), DDIs will be reassessed from Step 4. New findings will be presented to and discussed with the expert panel. Step 4 and Step 5 will be focussed on whether earlier decisions will be affected by new evidence or changes in medical practice.
3. Healthcare providers can initiate a (re)assessment when medical practice will evolve.

## Ethics

As there will be no patients nor data directly derived from patients involved in our methodology, this protocol was not evaluated by an ethics committee. Also, there are no privacy issues with respect to study data applicable.

## Anticipated results

Three examples of the classification of a probe P-gp-inducer, -inhibitor and -substrate are given, according to the assessment reports generated in Step 4. We will also describe the conclusions on whether the classified substrate will be involved in clinically relevant P-gp-interacties (yes or no). Finally, we will describe the added value of our approach in comparison to official product information and other sources.

Example 1 Apalutamide as P-gp-inducer.

According to the FDA apalutamide is a strong CYP3A4-inducer and the manufacturer suggests activation of PXR (FDA, 2024; Erleada^®^ apalutamide, 2024). Following the concepts and definition (step 2), apalutamide is evaluated for an effect on P-gp. Using the PubMed and Embase search strategy for “modulator” shown in Table 1, additional evidence was found with the probe-substrate fexofenadine (AUC x0.7) (Duran et al., 2020). According to Table 2, apalutamide will be classified as an established P-gp-inducer.

Example 2 Cyclosporine as P-gp-inhibitor.

Following step 3, cyclosporine is evaluated as a P-gp-inhibitor by using the PubMed and Embase search strategy for “modulator” shown in Table 1. Clinical data with digoxin (increased plasma concentration), edoxaban (AUC x1.7) and aliskiren (AUC x5) show a significant increase in exposure of these probe-substrates when co-administered with cyclosporine (Parasrampuria et al., 2016; Rebello et al., 2011; Dorian et al., 1988). It should be noted that cyclosporine is also known to be an OATP-inhibitor and a weak CYP3A4-inhibitor. Aliskiren is likely to be an OATP2B1-substrate but is only minimally metabolized by CYP3A4 (FDA, 2024; Rebello et al., 2011; Rasilez^®^ aliskiren, 2023). It is likely that the increase in aliskiren AUC caused by cyclosporine is mainly due to P-gp rather than OATP2B1 inhibition, as cyclosporine is not a strong inhibitor of this specific OATP-transporter (Rebello et al., 2011). In addition, there is evidence with other (probe-)P-gp-substrates that is sufficient to classify cyclosporine as an established P-gp-inhibitor according to Table 2.

Example 3 Fexofenadine as P-gp-substrate.

For the evaluation of fexofenadine as a P-gp-sustrate, the PubMed and Embase search strategy for for “substrate” shown in Table 1 was used. Clinical data with quinidine showed a significant increase in fexofenadine exposure by a factor of 2 (Bosilkovska et al., 2014). Significant decreases in fexofenadine exposure were seen with apalutamide (fexofenadine AUC x0.7) and carbamazepine (fexofenadine AUC x0.4-0.6) and increases in oral clearance were seen with rifampicin (fexofenadine oral clearance x2-3) (Duran et al., 2020; Yamada et al., 2009; Akamine et al., 2012; Hamman et al., 2001). It should be noted that the interpretation of the apparent effect of P-gp with rifampicin is complicated because fexofenadine is also known to be an OATP-substrate and rifampicin is known to modulate OATP. According to Table 2, fexofenadine will be classified as an established P-gp-substrate. The clinical relevance of the increased exposure of fexofenadine with P-gp-inhibitors is not considered relevant because the increased exposure of fexofenadine is within the range of plasma levels obtained in adequate and well-controlled clinical trials. The clinical relevance of the decreased exposure of fexofenadine with inducers is unclear. According to the criteria for assessing the risk of toxicity or reduced treatment efficacy of P-gp-drug-substrates (see flow chart in Supplementary Material 2), fexofenadine will be classified as high risk ‘no’.

### Clinically relevant P-gp mediated DDIs

The conclusions on clinically relevant P-gp-interactions (yes or no) are based on the combination of the assessment reports of the classified P-gp-substrates, which includes the assessment of clinical relevance and risks, with the classified P-gp-modulators. The initial conclusions on the clinical relevance of (potential) DDIs, as presented to the multidisciplinary expert panel, are presented in Table 3.

**TABLE 3:** Possible outcomes of P-gp-substrates with P-gp-inducers and -inhibitors, assessed for clinical relevance.

Established P-gp substrates		Inducers		Inhibitors
clinically relevant effect(s) with P-gp-inducer(s) and/or -inhibitor(s)?	high risk P-gp-drug-substrate?	Established P-gp-inducers	Predicted P-gp-inducers	Established P-gp-inhibitors
Yes or uncertain for inducers and/or inhibitors	Yes	Yes	Yes	Yes
Yes for inducers and/or inhibitors	No	Yes	No	Yes^a^
No for inducers and/or inhibitors	Yes	No	No	No
No or uncertain for inducers and/or inhibitors	No	No	No	No

^a^ clinically relevant DDIs, only for combinations of substrates with inhibitors with evidence of clinically relevant effects.

A distinction is made between *established P-gp-substrates* assessed as high risk “yes” and *established P-gp-substrates* assessed as high risk “no”. All combinations of *high risk P-gp-drug-substrates* with *established modulators* will be presented as clinically relevant DDIs. Exceptions will be made where specific combinations will be considered to be “not relevant”. Combinations of *high risk P-gp-drug-substrates* with *predicted P-gp-inducers* will also be presented as clinically relevant DDIs, in the context of the risks. These conclusions on the clinical relevance of (potential) DDIs are different for *P-gp-substrates* not classified as *high risk*: only combinations of *P-gp-substrates* that are assessed as high risk “no” with *predicted P-gp-inducers and P-gp-inhibitors* with evidence for clinically relevant effects will be considered to be relevant. Hence in the context of the risk, extrapolation to potential DDIs with these substrates and *predicted P-gp-inducers* will be considered to be irrelevant. In addition, DDIs with P-gp-inhibitors have shown limited effects, hence only combinations with *high risk substrates* and combinations of substrates and inhibitors with evidence for relevant effects will be considered to be clinically relevant (Umeyama et al., 2014).

All other combinations of P-gp-modulators with substrates not classified as established, for example, when the robustness of the evidence for substrates is unclear, will be considered to be not clinically relevant DDIs.

In all situations, study outcomes for inducer/inhibitor-substrate combinations will be discussed with the expert panel.

According to Table 3, the concluding recommendations for the example fexofenadine as an established *P-gp-substrate with high risk ‘no’* are that DDIs with established inhibitors and inducers are not considered clinically relevant due to no and uncertain clinical relevance, respectively.

### Comparison with official product information and other databases

As described in Step 6, the final recommendations will be published in a separate article. In addition, we will be able to compare how different sources describe specific drugs as inducer, inhibitor or substrate. Comparisons will be made with information presented in the official product information (SmPCs and FDA drug labels), with frequently used sources of information on DDIs such as Stockley’s and Up-to-date and with previous reviews. This comparison will allow to show the differences and identify medications that are currently overlooked as P-gp modulators in existing sources, as well as medications that are associated with P-gp without convincing clinical relevance according to the results of our protocol.

## Discussion

We have developed a protocol to assess changes in systemic drug exposure due to DDIs involving P-gp (P-glycoprotein). The application of this protocol will generate practice recommendations and alerts for clinically relevant DDIs, as well as rationales for DDIs that will be considered irrelevant. A combination of literature review and expert opinion will classify potential P-gp-modulators and -substrates and provide conclusions on which combinations of classified modulators and substrates will lead to clinically relevant DDIs. This approach will provide healthcare professionals with more validated information, in addition to other existing sources such as SmPCs, to support them to make consistent decisions on how to manage P-gp mediated DDIs.

A combined literature review and expert opinion approach has helped healthcare professionals interpret complex therapeutic challenges (van Leeuwen et al., 2022; Wang et al., 2019; Weersink et al., 2016; van Tongeren et al., 2020; van Roon et al., 2005). A similar approach seems feasible, but some challenges to evaluate the evidence regarding P-gp mediated DDIs require a more pragmatic approach. Although P-gp is a transporter involved in well-known DDIs, there is no standardized approach for translating investigational DDI studies with P-gp involvement into specific practice recommendations. In addition, classifying investigational drugs as modulators or substrates of P-gp and/or listing potential P-gp mediated DDIs is complicated because there is currently no classification system for P-gp as there is for DDIs involving CYP-enzymes (ICH, 2022; Elmeliegy et al., 2020; Burger et al., 2023; Henderson et al., 2021).

From a pharmacological perspective, this approach is challenging as accurate interpretation of the apparent effect of P-gp is complicated by overlap with other transporters and drug metabolizing enzymes, particularly CYP3A4 (ICH, 2022; Zamek-Gliszczynski et al., 2021; Elmeliegy et al., 2020; Lin and Yamazaki, 2003; Biotechnology, 2024; Tornio et al., 2019; Zhang et al., 2018). As the number of selective P-gp-modulators and -substrates is limited, interpretation of the apparent P-gp effect may not always be possible. For example, the apparent effect of P-gp cannot be concluded for (potential) P-gp-substrates that are also metabolized by CYP3A4 and have been assessed only with dual modulators of P-gp and CYP3A4 in clinical studies. In this case, there are no data on the effect of P-gp without the influence of CYP3A4, so these substrates will not be included as clinically relevant P-gp substrates. However, these drug combination will result in a DDI within the established classification system of CYP3A4, as the effect of P-gp is less than that of CYP3A4 (ICH, 2022; Elmeliegy et al., 2020; Umeyama et al., 2014). Another example where it is important to check that potential substrates are not also substrates for OATP or BCRP is in DDI studies with cyclosporine, a known inhibitor of these transporters.

Despite the challenges of assessing the evidence for apparent P-gp effects in DDIs, this approach goes beyond that of a previous review by developing transparent lists of evaluated P-gp-modulators and -substrates, including recommendations with a focus on clinical practice (Lund et al., 2017).

Many SmPCs report data on DDIs involving P-gp, but recommendations for clinical practice are often inconclusive, inconsistent and/or not always up to date or based on the latest evidence (Henderson et al., 2021). For example, the SmPC for talazoparib states that it may be a clinically relevant P-gp-substrate, despite negative evidence of a DDI with rifampicin. Our systematic approach will assess whether practice recommendations are needed for combinations of talazoparib with other P-gp-inducers, or whether the lack of effect of rifampicin on talazoparib exposure can be extrapolated to other P-gp-inducers (Henderson et al., 2021; Talzenna^®^ talazoparib, 2024). Another example is the SmPC for capmatinib, which states that caution should be exercised when co-administered with P-gp-substrates. However, edoxaban, for example, is not included in this warning as there is no general list of common relevant P-gp-substrates (Prescribing Information Tabrecta^®^ capmatinib, 2023). This interaction, given the potential consequences, may be considered as clinically relevant DDI (Otten et al., 2022). Another interesting example is enzalutamide, which will be evaluated as both a P-gp-inducer and -inhibitor based on its known CYP3A4-inducing effects through PXR-induction and a human *in vivo* study showing P-gp-inhibitory effects when administered simultaneously with digoxin. Our systematic approach will assist pragmatic interpretation and recommendations about such complex evidence DDIs.

Therefore, the present protocol may fill the current gap in the official product information for P-gp-related DDIs that have not been studied, based on extrapolation where possible (Burger et al., 2023). This protocol may also be applied to P-gp-activators, substances that enhance the transport function of P-gp without increasing its expression. Although no P-gp-activators are currently marketed as medicines (i.e., thioxanthone-5), they may be in the future. When available, P-gp-activators can be added to the list of established P-gp-inducers and included in DDIs with established P-gp-substrates. However, for practice recommendations, it should be noted that the onset and offset of action of P-gp-activators is likely to be different from that of P-gp inducers, as they differ from P-gp-inducers in that they immediately increase P-gp transport activity without interfering with P-gp protein expression (Veiga-Matos et al., 2023).

A strength of the present study is that it will combine literature review and expert opinion to generate practical recommendations for clinical practice. For DDIs where limited or no data are available, conclusion might be reached by expert-opinion and extrapolation, based the pharmacological characteristics of these drugs.

Improving drug safety while minimising the risk of alert fatigue is a challenge. In this context, we will narrow the focus on those DDIs that are most likely to be clinically relevant. For example, as the clinical relevance of DDIs caused by P-gp inhibition alone has been shown to be limited, only combinations with P-gp-drug-substrates classified as *high risk* or with evidence of clinically relevant effects will require practice recommendations (Umeyama et al., 2014). We believe that the critical evaluation of the robustness of the evidence, the clinical relevance of the DDI-findings and whether the substrate poses a high risk of toxicity or reduced treatment efficacy, will contribute to balanced practice recommendations. Hence, this approach will result in sensitive and specific alerts and practical recommendations, thereby limiting alert fatigue among healthcare professionals (van der Sijs et al., 2006; Phansalkar et al., 2013). Finally, the results will be implemented in a national CDSS for healthcare professionals providing alerts at the point of care. This will improve pharmaceutical care for the individual patient.

Obviously, our design also has certain limitations. First, we choose to focus on inhibition of P-gp in the intestine, and not renal or hepatic P-gp, or P-gp at the blood brain barrier (BBB). Although most of the current knowledge about changes in systemic drug exposure relates to intestinal P-gp, we cannot rule out that future studies will provide more information about the clinical relevance of other sites (e.g., BBB) (Zamek-Gliszczynski et al., 2021; Elmeliegy et al., 2020; Kalvass et al., 2013). Second, our focus is on evidence from human *in vivo* studies, as it is difficult to translate *in vitro* studies to clinical relevance (ICH, 2022; FDA, 2020b; EMA, 2012; Kido et al., 2024). The number of clinical DDI studies that investigate P-gp as mechanism is limited and we expect that some of the studies that have been published will be fragmented or incomplete in terms of information to assess the actual effect on P-gp. Therefore, in case of limited or inconclusive clinical evidence *in vitro* data may be included to provide a mechanistic understanding and the final recommendations will be based on the clinical experience of the multidisciplinary expert panel and adopted by consensus. The considerations and discussions within the expert panel will be included in the assessment reports. Finally, the outcomes will only be implemented in Dutch CDSS for healthcare professionals. Therefore, publishing the results in international journals for dissemination of pharmacological knowledge concerning DDIs with P-gp as underlying mechanism is warranted.

The practice recommendations resulting from this protocol will improve clinical practice by providing guidance on managing specific combinations of P-gp mediated drugs in individual patients. However, these recommendations are based on the pharmacological properties of the medications and need to be tailored to each patient according to their individual characteristics. Consequently, healthcare providers have to consider additional factors such as co-morbidities, specific conditions (e.g., pregnancy), age, genetics, and altered pharmacokinetics due to impaired kidney/liver function or altered pharmacokinetics due to obesity or bariatric surgery (Diesveld et al., 2021; Weersink et al., 2018; Abdullah-Koolmees et al., 2020; Ashley and Dunleavy, 2019; Bories et al., 2021). All of this should be in accordance with accepted clinical standards. This requires a professional assessment, as advanced CDSS involving complex interactions, such as DDIs in patients with specific comorbidities or drug-drug-gene interactions, are not yet widely used (Pasternak et al., 2023; Busa et al., 2018; Yeung et al., 2015). The professional’s advice will be a clinical decision derived from multiple sources of information, sometimes supplemented by alerts from CDSS. Although CDSS are becoming more advanced through the integration of knowledge bases, patient data, big data, and artificial intelligence, complex clinical questions regarding drug interactions will still require careful professional judgment and appropriate monitoring of patients once therapy has started (Graafsma et al., 2024).

In addition, drug-drug-gene interactions for transporters are less well studied than for metabolizing enzymes (Malki and Pearson, 2020). Despite evidence for (inter-individual) variability in ABCB1 gene expression and P-gp function, the genetic contribution to drug PK is variable and conflicting (Saiz-Rodríguez et al., 2018; Hodges et al., 2024). More knowledge on this topic is needed to assess the pharmacogenetic effect in relation to DDIs involving P-gp (Hodges et al., 2024; Lenoir et al., 2022; PharmVar and Pharmacogene Variation Consortium, 2024).

Finally, this study will result in recommendations for gaps in existing knowledge about P-gp-mediated DDIs by the extrapolation of conclusions for DDIs where limited or no data are available. Analysis of these gaps will help prioritizetargets for future clinical studies. For example, to design studies that focus more on identifying the underlying pharmacological mechanisms in order to more specifically identify potential DDIs and studies to assess outcomes such as the actual occurrence of DDIs or the avoidance of DDIs (Henderson et al., 2021). In addition, it may be possible for registration authorities to collect real-world evidence after registration to evaluate these potential DDIs through cohort studies or analysis of spontaneously reported ADRs (Zhu et al., 2023; Mofid et al., 2022; Bakker et al., 2023). This systematic protocol may also be useful for the assessment of DDIs with other underlying mechanisms. To ensure quality and consistency in DDI management, it is important that the approach for assessing (potential) DDIs and developing practice recommendations is standardized and transparent (ICH, 2022; Floor-Schreudering et al., 2014).

In conclusion, this protocol describes a standardized, evidence- and expert opinion-based assessment of P-gp-mediated DDIs with respect to the clinical significance of changes in systemic drug exposure. This approach will provide practical recommendations for the management of relevant P-gp mediated DDIs, as well as transparent rationales for DDIs that are considered to be irrelevant. This knowledge will be implemented directly in a national CDSS for healthcare professionals. These recommendations will improve individual patient care by supporting healthcare professionals to make consistent decisions on how to manage P-gp mediated DDIs. Finally, this protocol can identify gaps in existing knowledge that will form a research agenda for future investigations.

## Abbreviations

ABCB1, adenosine triphosphate (ATP) binding cassette sub-family B member 1 transporter; CDS(S), Clinical decision support (system); DDI, Drug-drug interaction; EMA, European Medicine Authority; EPAR, European Public Assessment Report; FDA, Food and Drug Administration; ICH, International Council for Harmonisation of Technical Requirements for Pharmaceuticals for Human Use; P-gp, P-glycoprotein; MDR1, multidrug resistance protein; S(m) PC, Summary of Product Characteristics; TDM, therapeutic drug monitoring.
