# Supplementary material for: A practical assessment protocol for clinically relevant P-glycoprotein-mediated drug-drug interactions
Source: Front Pharmacol. 2024 Sep 30;15:1412692. doi: 10.3389/fphar.2024.1412692 (PMC11472019; doi:10.3389/fphar.2024.1412692)
Supplement: Supplementary file 1 [file DataSheet1.pdf]

Supplementary Material

A practical assessment protocol for clinically relevant P-glycoprotein-mediated drug-drug interactions

Leonie Bogaard[1], Kayan Tsoi[1], Bas van de Steeg[2], Esther F.A. Brandon[3], Lisanne Geers[4], Margreet van Herwaarden[5], Frank Jansman[6,7], Dominique Maas[8], Margje Monster-Simons[3,9], David S.Y. Ong[10,11], Sander D. Borgsteede[1*]

[1]Dept. Clinical Decision Support, Health Base Foundation, Houten, the Netherlands.
[2]Department of Clinical Pharmacy, Canisius Wilhelmina Hospital, Nijmegen, The Netherlands.
[3]Dutch Medicines Evaluation Board, Utrecht, The Netherlands.
[4]Department of Clinical Pharmacy, Rijnstate Hospital, Arnhem, The Netherlands.
[5]Pharmacist, Groesbeek, Netherlands.
[6]Unit of PharmacoTherapy, Epidemiology and Economics, Groningen Research Institute of Pharmacy, University of Groningen, Groningen, Netherlands.
[7]Department of Clinical Pharmacy, Deventer Teaching Hospital, Deventer, the Netherlands.
[8]Department of Internal Medicine, Radboud university medical center, Nijmegen, The Netherlands.
[9]Department of Clinical Pharmacy and Pharmacology, University of Groningen, Groningen, The Netherlands.
[10]Department of Medical Microbiology and Infection Control, Franciscus Gasthuis & Vlietland Hospital, Rotterdam, The Netherlands.
[11]Department of Epidemiology, Julius Center for Health Sciences and Primary Care, University Medical Center Utrecht, Utrecht University, Utrecht, The Netherlands.

* Correspondence: Sander Borgsteede, sander.borgsteede@healthbase.nl

Supplement 1 2
Flow chart of the seven steps in this protocol. 2
Supplement 2 3
Flow chart to assess the risk of toxicity or reduced treatment efficacy of P-gp-drug-substrates. . 3
Supplement 3 4
Standardized assessment reports for inducers, inhibitors and substrates. 4
Supplement 4 11
PRISMA-P (Preferred Reporting Items for Systematic review and Meta-Analysis Protocols) 2015 checklist. 11

Supplement 1

Flow chart of the seven steps in this protocol.

STEP 1, setting up an expert panel.
Outcome:
- Fixed expert panel.

↓

STEP 2, concepts and definitions.
Outcomes:
- General assumptions for P-gp-mediated DDIs.
- Definition of potentially clinically relevant effects.

↓

STEP 3, selection of drugs to be evaluated.
Outcome:
- Potential P-gp-inducers, inhibitors and substrates to be evaluated.

↓

STEP 4, selection and presentation of evidence.
Outcomes:
- Search strategy and literature selection criteria.
- Assessment reports with preliminary conclusions on the classification of potential P-gp-modulators and -substrates, including preliminary conclusions on potentially clinically relevant effects.

↓

STEP 5, conclusion and practice recommendations for clinical practice.
Outcomes:
- Conclusion on classifications
- Conclusions on clinical relevance and practice recommendations

↓

STEP 6, implementation in electronic prescribing and dispensing systems.
Outcome:
- Publish recommendations in Dutch guidelines for DDIs (CM) and in CDSS

↓

STEP 7, updating.
Outcome:
- New assessment reports and recommendations
- Update of existing assessment reports and recommendations

DDIs = Drug-drug interactions, CM = Commentaren Medicatiebewaking, CDSS = Clinical decision support system.

Supplement 2

Flow chart to assess the risk of toxicity or reduced treatment efficacy of P-gp-drug-substrates.

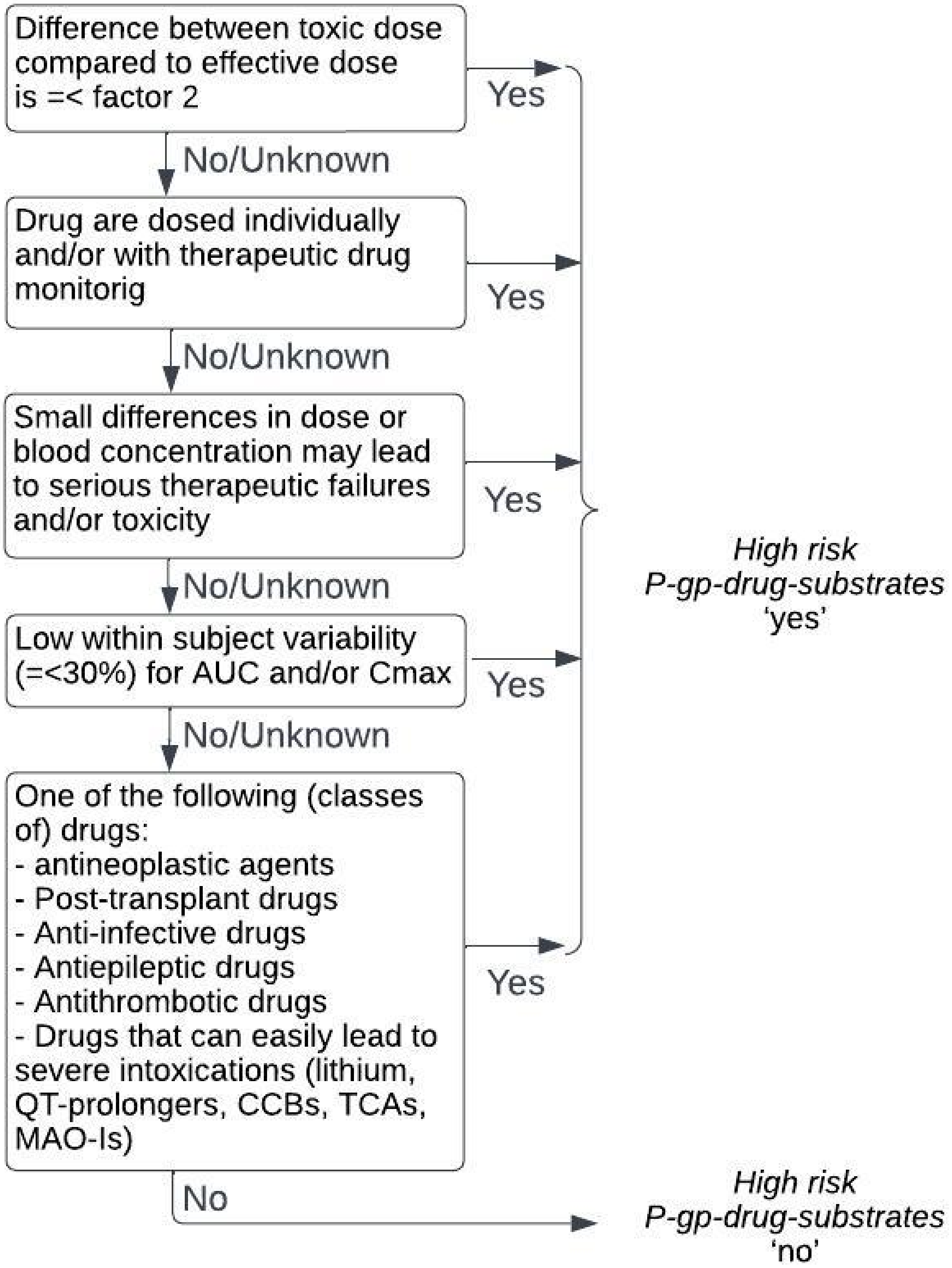

AUC = Area under the curve, C_{max} = maximum concentration, CCBs = Calcium channel blockers, TCAs = Tricyclic antidepressants, MAOIs = Monoamine Oxidase Inhibitors.

Supplement 3

Standardized assessment reports for inducers, inhibitors and substrates.

Assessment report for p-gp-inducers and inhibitors

Status:
Date last search:
Abbreviations:
PXR: Pregnane X receptor. **ABCB1**: ATP Binding Cassette Subfamily B Member 1, a Protein Coding gene. **MDR1:** multidrug resistance protein 1, also known as P-glycoprotein.

Conclusion	Classification#	Clarification
Comments		

#

Classification	Description
P-gp-inducer, established	Evidence of inhibiting/inducing-effect in human in vivo studies with P-gp probe substrate(s), i.e. 0.8 > $AUCR_{probe\text{-}substrate}$ ≥ 1.25.
P-gp-inducer, predicted	Established strong/moderate CYP3A4-inducers, but lack of human in vivo studies with P-gp probe substrates that will classify them as established or as not relevant
P-gp- inducer, unclear	inconclusive evidence from human in vivo studies with P-gp probe substrate(s), e.g. where some combinations of modulator and probe-substrate result in an interaction-effect, while no effect with other probe-substrate
P-gp- inducer, lack of evidence	Lack of evidence from human in vivo studies.
P-gp-inducer, not relevant	Lack of inducing/inhibiting-effects in human in vivo studies with p-g-probe-substrates, i.e. 0.8 ≤ $AUCR_{probe\text{-}substrate}$ < 1.25
P-gp-inhibitor, established	Evidence of inhibiting/inducing-effect in human in vivo studies with P-gp probe substrate(s), i.e. 0.8 > $AUCR_{probe\text{-}substrate}$ ≥ 1.25.
P-gp-inhibitor, unclear	Inconclusive evidence from human in vivo studies with P-gp probe substrate(s), e.g. where some combinations of modulator and probe-substrate result in an interaction-effect, while no effect with other probe-substrate
P-gp-inhibitor, lack of evidence	Lack of evidence from human in vivo studies.
P-gp-inhibitor, not relevant	Lack of inducing/inhibiting-effects in human in vivo studies with p-g-probe-substrates, i.e. 0.8 ≤ $AUCR_{probe\text{-}substrate}$ < 1.25

Summary

Probe-Substraat	Effect	Evidence summary and mechanism
Digoxine		
Dabigatran		
Edoxaban		
Fexofenadine		
Aliskiren		
talinolol		

SPC/ EPAR

	Intervention substrates+dose	Time	Effect		
			AUC	Cmax	Clinical effect/ AE/study type
In vitro study			In factor		
In vivo study					

FDA

	Intervention substrates+dose	time	Effect		
			AUC	Cmax	Clinical effect/ AE
In vitro study			In factor		
In vivo study					

EMA clinical data:[https://clinicaldata.ema.europa.eu/web/cdp/home]

Additional studies (in-vivo)

PUBMED

("inducer"[mesh] OR "inducer"[TIAB]) AND ("Dabigatran"[Mesh] OR "Dabigatran"[TIAB] OR "edoxaban"[Supplementary Concept] OR "edoxaban"[TIAB] OR "Digoxin"[Mesh] OR "Digoxin"[TIAB] OR "fexofenadine" [Supplementary Concept] OR "fexofenadine" [TIAB] OR "talinolol" [Supplementary Concept] OR "talinolol" [TIAB] OR "aliskiren" [Supplementary Concept] OR "aliskiren" [TIAB]) Filters: Humans AND clinical study AND case reports

("inducer" OR "inducer"[TIAB]) AND ((ATP Binding Cassette Transporter, Subfamily B, Member 1[Mesh]) OR (P-Glycoprotein[TIAB]) OR (P Glycoprotein[TIAB]) OR (P-gp[TIAB]) OR (ABCB1[TIAB]) OR (MDR1[TIAB])) Filters: Humans AND clinical study AND case reports

EMBASE

inducer/mj AND ('dabigatran'/mj OR 'edoxaban'/mj OR 'digoxin'/mj OR 'fexofenadine'/mj OR 'talinolol'/mj OR 'aliskiren'/mj)

inducer/mj AND ('abc transporter subfamily b'/mj OR 'multidrug resistance protein 1'/mj)

Reference	Intervention substrates+dose	Level of evidence (LOE) Pharmacokinetic effects on substrate		Clinical effect/ AE
[cite]<br><br>[study type]		LOE		
		Time		
		AUC		
		Cmax		

**OCEBM Levels of Evidence Working Group. The Oxford 2011 Levels of Evidence. Oxford Centre for Evidence-Based Medicine; 2011. Level may be graded down on the basis of study quality, imprecision, indirectness (study PICO does not match questions PICO), because of inconsistency between studies, or because the absolute effect size is very small; Level may be graded up if there is a large or very large effect size.*

Additional studies (in vitro)

Pubmed:<br>("inducer" OR "inducer"[TIAB]) AND ((ATP Binding Cassette Transporter, Subfamily B, Member 1[Mesh]) OR (P-Glycoprotein[TIAB]) OR (P Glycoprotein[TIAB]) OR (P-gp[TIAB]) OR (ABCB1[TIAB]) OR (MDR1[TIAB])) AND "in vitro"<br><br>Embase:<br>inducerr/mj AND ('abc transporter subfamily b'/mj OR 'multidrug resistance protein 1'/mj)		
Reference	Intervention substrates+dose	Clinical effect/ AE/study type

Stockley's Drug Interactions

Baxter K (ed). Stockley's Drug Interactions [online]. London: Pharmaceutical Press. www.medicinescomplete.com<br><br>Title<br>Last modification	monograph<br>Check for (new) studies; not found by pubmed/embase?

Guidelines

Source	Results
EHRA	
Hansten	
SPC's probe substrates	
KNMP G-standaard	Monograph: [titel]
Relevance	Yes/No
Risk analysis	

Lund et al. (2017)

classification as P-glycoprotein-inducer [in vitro/clinical relevance in vivo*]	Remarks
P-gp-Inducer: [/]	Refers to

*P-gp modulation (in vitro) – Clinical relevance (in vivo). y=yes u=uncertain.

Additional information

Mechanism P-gp induction [PXR/ CAR]	
CYP3A4- inducer?	
Discussion with expert panel	

Assessment report for p-gp-substrates: [substrate]

Review date:

Abbreviations:

PXR: Pregnane X receptor. **ABCB1**: ATP Binding Cassette Subfamily B Member 1, a Protein Coding gene. **MDR1:** multidrug resistance protein 1, also known as P-glycoprotein.
[Grey cells = no information available]

Conclusion	Classification	Clarification
	#	With p-gp-inducers:<br>With P-gp-inhibitors:
Clinically relevant	[yes / no / uncertain]	based on pharmacokinetic evidence (e.g. AUC/Cmax) and safety evidence (toxicity/ reduced treatment efficacy) from studies
High risk	[yes / no]	toxic doses vs normal dose <factor 2?<br>dosed individually?<br>doubling the dose with<br>increased risks of toxicity?<br>small intra-individual differences?<br>drugs belong to cytostatics/transplant/etc.?
Role of CYP in metabolism		
Comments		

#

Classification	**Description**
P-gp-substrate, established	Evidence from human in vivo studies of P-gp-inducing OR inhibiting effects on the potential substrate, i.e. $0.8 > AUCR_{substrate} \geq x1.25$.
P-gp-substrate, unclear	Inconclusive evidence from human in vivo studies with the selection of P-gp-inducers, e.g. where some combinations of the potential substrate with inducer result in an interaction-effect, while no effect with other inducers. The same applies for potential substrates with the selection of P-gp-inhibitors.
P-gp-substrate, lack evidence	Lack of evidence from human in vivo studies with the selection of P-gp-inducers and inhibitors.
P-gp-substrate, not relevant	Lack of P-gp-inducing or inhibiting effects on the potential substrate,<br>i.e. $0.8 \leq AUCR_{potential\text{-}substrate} < 1.25$ or

Summary

Probe-inducer/inhibitor	Effect	Evidence summary and mechanism

SPC/ EPAR

	Which inducer/inhibitor+ dose	Time	Effect		
			AUC	Cmax	Clinical effect/ AE/study type
In vitro study			In factor		
In vivo study					

FDA

	Which inducer/inhibitor+ dose	time	Effect		
			AUC	Cmax	Clinical effect/ AE
In vitro study			In factor		
In vivo study					

EMA clinical data:[https://clinicaldata.ema.europa.eu/web/cdp/home]

Additional studies

Additional studies (in-vivo) with inducers

PUBMED

(substrate[Mesh] OR substrate[TIAB]) AND (rifampin[Mesh] OR rifampicin[TIAB] OR Carbamazepine[Mesh] OR Carbamazepine[TIAB] OR Phenytoin[Mesh] OR Phenytoin[TIAB] OR apalutamide[Supplementary Concept] OR apalutamide[TIAB] OR efavirenz[Supplementary Concept] OR efavirenz[TIAB] OR Hypericum[Mesh] OR Hypericum[TIAB]) Filters: Humans AND clinical study AND case reports

(substrate[Mesh] OR subsrate[TIAB]) AND ((“ATP Binding Cassette Transporter, Subfamily B, Member 1” [Mesh]) OR (P-Glycoprotein[TIAB]) OR (“P Glycoprotein”[TIAB]) OR (P-gp[TIAB]) OR (ABCB1[TIAB]) OR (MDR1[TIAB])) Filters: Humans AND clinical study AND case reports

EMBASE

substrate/mj AND ('rifampicin'/mj OR 'carbamazepine'/mj OR 'phenytoin'/mj OR 'apalutamide'/mj OR 'efavirenz'/mj OR 'Hypericum perforatum'/mj)

substrate/mj AND ('abc transporter subfamily b'/mj OR 'multidrug resistance protein 1'/mj)

Reference	Intervention substrates+dose	Level of evidence (LOE) Pharmacokinetic effects on substrate		Clinical effect/ AE
[cite]<br><br>[study type]	Middel(en), dosis en tijdslijn	LOE*		
		Time		
		AUC		
		Cmax		

**OCEBM Levels of Evidence Working Group. The Oxford 2011 Levels of Evidence. Oxford Centre for Evidence-Based Medicine; 2011. Level may be graded down on the basis of study quality, imprecision, indirectness (study PICO does not match questions PICO), because of inconsistency between studies, or because the absolute effect size is very small; Level may be graded up if there is a large or very large effect size.*

Additional studies (in-vivo) with inhibitors

PUBMED

("substrate"[Mesh] OR " substrate "[TIAB]) AND ("Amiodarone"[Mesh] OR "Amiodarone"[TIAB] OR "ranolazine"[Mesh] OR "ranolazine"[TIAB] OR "Quinidine"[Mesh] OR "Quinidine"[TIAB] OR "Propafenone"[Mesh] OR "Propafenone"[TIAB] OR "Cyclosporine"[Mesh] OR "Cyclosporine"[TIAB]) Filters: Humans AND clinical study AND case reports

("substrate"[Mesh] OR "substrate"[TIAB]) AND ((“ATP Binding Cassette Transporter, Subfamily B, Member 1/antagonists and inhibitors” [Mesh]) OR (P-Glycoprotein[TIAB]) OR (P Glycoprotein[TIAB]) OR (P-gp[TIAB]) OR (ABCB1[TIAB]) OR (MDR1[TIAB])) Filters: Humans AND clinical study AND case reports

EMBASE

substrate/mj AND ('amiodarone' OR ranolazine/mj OR 'propafenone'/mj OR 'quinidine'/mj OR ‘cyclosporine’/mj)

substrate/mj AND ('abc transporter subfamily b'/mj OR 'multidrug resistance protein 1'/mj)

Reference	Intervention substrates+dose	Level of evidence (LOE) Pharmacokinetic effects on substrate		Clinical effect/ AE
[cite]<br><br>[study type]		LOE		
		Time		
		AUC		
		Cmax		

Additional studies (in vitro)

Pubmed:

("substrate"[Mesh] OR " substrate "[TIAB]) AND ((“ATP Binding Cassette Transporter, Subfamily B, Member 1” [Mesh]) OR (P-Glycoprotein[TIAB]) OR (P Glycoprotein[TIAB]) OR (P-gp[TIAB]) OR (ABCB1[TIAB]) OR (MDR1[TIAB])) AND “in vitro”

Embase:

substrate /mj AND ('abc transporter subfamily b'/mj OR 'multidrug resistance protein 1'/mj)

Reference	Intervention substrates+dose	Clinical effect/ AE/study type

Stockley's Drug Interactions

Baxter K (ed). Stockley's Drug Interactions [online]. London: Pharmaceutical Press. www.medicinescomplete.com<br><br>Title<br>Last modification	monograph<br>Check for (new) studies; not found by pubmed/embase?

Guidelines

Source	Results
Otten et al.	
Leeuwen et al.	
Handbook Hansten	
SPC's probe inh/ind	
KNMP G-standaard	Monograph: [titel]
Relevance	Yes/No
Risk analysis	

Lund et al. (2017)

classification as P-glycoprotein-substrate [in vitro/clinical relevance in vivo*]	Remarks
P-gp-substrate: [/]	Refers to

*P-gp modulation (in vitro) – Clinical relevance (in vivo). y=yes u=uncertain.

Additional information

CYP3A4 – substrate?	
Discussion with expert panel	

Supplement 4

PRISMA-P (Preferred Reporting Items for Systematic review and Meta-Analysis Protocols) 2015 checklist.

Section and topic	Item No	Checklist item	Reported on page(s) #
**ADMINISTRATIVE INFORMATION**			
Title:			
Identification	1a	Identify the report as a protocol of a systematic review	1
Update	1b	If the protocol is for an update of a previous systematic review, identify as such	NA
Registration	2	If registered, provide the name of the registry (such as PROSPERO) and registration number	NA
Authors:			
Contact	3a	Provide name, institutional affiliation, e-mail address of all protocol authors; provide physical mailing address of corresponding author	1
Contributions	3b	Describe contributions of protocol authors and identify the guarantor of the review	10
Amendments	4	If the protocol represents an amendment of a previously completed or published protocol, identify as such and list changes; otherwise, state plan for documenting important protocol amendments	NA
Support:			
Sources	5a	Indicate sources of financial or other support for the review	10
Sponsor	5b	Provide name for the review funder and/or sponsor	NA
Role of sponsor or funder	5c	Describe roles of funder(s), sponsor(s), and/or institution(s), if any, in developing the protocol	NA
**INTRODUCTION**			
Rationale	6	Describe the rationale for the review in the context of what is already known	2,3
Objectives	7	Provide an explicit statement of the question(s) the review will address with reference to participants, interventions, comparators, and outcomes (PICO)	3
**METHODS**			
Eligibility criteria	8	Specify the study characteristics (such as PICO, study design, setting, time frame) and report characteristics (such as years considered, language, publication status) to be used as criteria for eligibility for the review	4,5 Table 2
Information sources	9	Describe all intended information sources (such as electronic databases, contact with study authors, trial registers or other grey literature sources) with planned dates of coverage	4, 5 Supplement 3
Search strategy	10	Present draft of search strategy to be used for at least one electronic database, including planned limits, such that it could be repeated	Table 1, Supplement 3

Study records:			
Data management	11a	Describe the mechanism(s) that will be used to manage records and data throughout the review	5,6
Selection process	11b	State the process that will be used for selecting studies (such as two independent reviewers) through each phase of the review (that is, screening, eligibility and inclusion in meta-analysis)	4,5,6
Data collection process	11c	Describe planned method of extracting data from reports (such as piloting forms, done independently, in duplicate), any processes for obtaining and confirming data from investigators	4,5,6
Data items	12	List and define all variables for which data will be sought (such as PICO items, funding sources), any pre-planned data assumptions and simplifications	3,4,5<br>Table 2
Outcomes and prioritization	13	List and define all outcomes for which data will be sought, including prioritization of main and additional outcomes, with rationale	4,5<br>Table 2
Risk of bias in individual studies	14	Describe anticipated methods for assessing risk of bias of individual studies, including whether this will be done at the outcome or study level, or both; state how this information will be used in data synthesis	5,6
Data synthesis	15a	Describe criteria under which study data will be quantitatively synthesised	NA
	15b	If data are appropriate for quantitative synthesis, describe planned summary measures, methods of handling data and methods of combining data from studies, including any planned exploration of consistency (such as I^2, Kendall's τ)	NA
	15c	Describe any proposed additional analyses (such as sensitivity or subgroup analyses, meta-regression)	NA
	15d	If quantitative synthesis is not appropriate, describe the type of summary planned	3,4,5<br>Table 2
Meta-bias(es)	16	Specify any planned assessment of meta-bias(es) (such as publication bias across studies, selective reporting within studies)	7,8
Confidence in cumulative evidence	17	Describe how the strength of the body of evidence will be assessed (such as GRADE)	4,5

NA= Not applicable

From: Moher D, Shamseer L, Clarke M, Ghersi D, Liberati A, Petticrew M, et al. Preferred reporting items for systematic review and meta-analysis protocols (PRISMA-P) 2015 statement. Syst Rev. 2015;4(1):1.

For more information, visit:www.prisma-statement.org
